# Secondary dissemination in children with high-grade malignant gliomas and diffuse intrinsic pontine gliomas

**DOI:** 10.1038/sj.bjc.6603402

**Published:** 2006-10-03

**Authors:** S Wagner, M Benesch, F Berthold, A K Gnekow, S Rutkowski, R Sträter, M Warmuth-Metz, R-D Kortmann, T Pietsch, J E A Wolff

**Affiliations:** 1 Department of Pediatric Hematology and Oncology, Klinik St Hedwig, University of Regensburg, Regensburg, Germany; 2 Division of Pediatric Hematology and Oncology, Department of Pediatrics and Adolescent Medicine, Medical University of Graz, Graz, Austria; 3 Department of Pediatric Hematology and Oncology, University of Köln, Köln, Germany; 4 Hospital for Children and Adolescents, Augsburg, Germany; 5 Department of Pediatric Oncology, Children's Hospital, University of Würzburg, Würzburg, Germany; 6 Department of Pediatric Hematology and Oncology, University Children's Hospital, Münster, Germany; 7 Department of Neuroradiology, University of Würzburg, Würzburg, Germany; 8 Department of Radiotherapy, University of Leipzig, Leipzig, Germany; 9 Department of Neuropathology, University of Bonn, Bonn, Germany; 10 Department of Pediatrics, The University of Texas MD Anderson Cancer Center, Houston, TX, USA

**Keywords:** secondary dissemination, leptomeningeal dissemination, high-grade gliomas, children

## Abstract

In children, treatment regimen for high-grade gliomas (HGG) and diffuse intrinsic pontine gliomas (DIPG) are generally not stratified according to disease stage. The hypothesis was that secondary disseminating disease (SDD) in children with HGG is related to an even worse outcome. Description of SDD pattern was performed. In total, 270 children with newly diagnosed HGG or DIPG were eligible for retrospective analysis of SDD. Medical and computer records of these patients were reviewed for demographic characteristics, sites of dissemination, prognostic variables. Forty-six (17%) of the 270 patients had developed SDD. The median time to SDD was 8.2 months. The median overall survival (OS) after dissemination was 3.2 months. The SDD was located parenchymal in the supratentorial (34.8%), infratentorial (6.5%), supratentorial and infratentorial (19.6%), spinal (10.9%), spinal and cerebral (6.5%) regions of the CNS, or leptomeningeal (21.7%). For HGG patients, the median OS was shorter among patients with SDD than among patients without SDD (1.02 *vs* 1.41 years, *P*=0.0495). In the group of patients with SDD, patients with cerebrospinal fluid dissemination had a worse outcome compared with patients with parenchymal metastases. Summarising, SDD is a negative prognostic factor for patients with HGG outside the pons. Treatment stratification should be considered.

Tumours of the central nervous system (CNS) are the most common solid neoplasms in children (Pollack, 1994). High-grade gliomas (HGG) (World Health Organization grade III and IV) constitute only 10–20% of childhood CNS tumours. In the German Database for HGG, about 40% of children were registered with diffuse intrinsic pontine glioma (DIPG). The diagnosis of DIPG is performed by radiological criteria: larger than 50% of the pontine diameter (Wagner *et al*, 2006) combined normally a short history of disease. All other criteria are not suitable to distinguish between focal and diffuse pontine glioma: DIPG includes tumours of WHO grade I-IV histology showing contrast enhancement on magnetic resonance imaging (MRI) and areas of necrosis and /or exophytic components.

Owing to the small number of children with these neoplasms, it is difficult to design and conduct prospective clinical trails of treatments for HGG. In contrast to other childhood malignancies, where treatment according to disease stage is standard, HGG patients with metastases are treated the same way as patients with localised disease. Owing to low incidences of secondary metastases in HGG and DIPG there is no impact for alternative treatment strategies.

In a previous study, we showed that about 3% of children with HGG or DIPG initially present with disseminated tumour spread at diagnosis so called primary dissemination of disease, stressing the need for a complete initial diagnostic workup, including neuroaxis MRI and cerebrospinal fluid (CSF) examination (Benesch *et al*, 2005). Most reports on tumour dissemination of HGG and DIPG after diagnosis, so called secondary dissemination of disease (SDD), are case reports or small series; few reports include large numbers of patients (Packer *et al*, 1985, 1990, 1993; Dropcho *et al*, 1987; Vertosick and Selker, 1990; Grabb *et al*, 1992; Arita *et al*, 1994; Heideman *et al*, 1997; Donahue *et al* 1998; Allen *et al*, 1999; Endo *et al*, 2003; Saito *et al*, 2003). These series mostly come from the late 1980s and early 1990s, when MRI was not routinely available for follow-up investigations (Awad *et al*, 1986; Vertosick and Selker, 1990; Arita *et al*, 1994). Secondary disseminating disease has been reported in 14–25% of adult patients with HGG or DIPG. The percentage of paediatric patients with SDD varies remarkably among studies (Table 1).

Data of patients with HGG and DIPG included into this retrospective study were recruited from a German HGG database in which patients were registered since 1995 in a prospective series of consecutive single-arm protocols using common diagnostic criteria for eligibility, with overall survival (OS) as the primary end point for comparison. The induction treatments consisted of conformal normal-fractionated radiotherapy and chemotherapy (Wolff *et al*, 2000, 2002a, 2002b, 2002c; Wagner *et al*, 2005), such as trofosfamide and oral etoposide (Dropcho *et al*, 1987; Vertosick and Selker, 1990); cisplatin, etoposide, ifosfamide (PEI) (Wolff *et al*, 2002b); or PEI plus vincristine or PEI plus high-dose methotrexate (Wagner *et al*, 2005). Data on metastatic disease were included in baseline information, but they did not alter the treatment strategies. In addition, all patients who developed disease relapse or progression were offered treatment according to the same protocol (Wagner *et al*, 2004). The most recent protocol treatment for patients with relapsed disease differed for those with completely *vs* incompletely resected recurrent disease, but the presence of metastases did not alter these treatment recommendations.

We performed a retrospective study in children with HGG or DIPG to determine, whether SDD is related to a worse outcome. Description of the pattern of SDD and the impact of SDD on OS comparing HGG with DIPG was a secondary objective; in order to obtain reliable data for treatment stratification in future studies.

## MATERIALS AND METHODS

Only patients with reliable radiographic and/or histopathologic evidence of SDD or non-SDD were included in this analysis. Eligibility criteria included informed consent of parents or legal guardians. Secondary disseminating disease was defined as the unequivocal presence of SDD outside the area of the primary tumour that had been documented by at least two different sources of information (e.g., two follow-up MRI studies) or proven by autopsy. Patients who were registered but who did not follow treatment guidelines were not excluded. All computer records and paper records were reviewed for diagnostic criteria of SDD by two investigators (MB, SW). Referring hospitals were contacted for missing data.

### Treatment

Patients of this study were recruited of a database in which patients were registered from July1995 until March 2005. Totally 653 patients were registered, and were enrolled into sequential protocols (A–D) of the study for HGG and DIPG of the Paediatric Oncology and Hematology Society of the German Language Group, initiated by JW. All protocols were reviewed and approved by Deutsche Krebsgesellschaft and the ethics committees of the participating centres.

Treatment protocols A (1995–1997), B (1997–1999), C (1999–2003) and D (has started 2003) have been previously described (Wolff *et al*, 2000, 2002a; Wagner *et al*, 2005). In all protocols, patients underwent conventional fractionated radiotherapy after surgery with a total dose of 59.6 or 54.0 Gy for children <6 years old and for children with brainstem gliomas. The volume of tissue irradiated was the tumour volume, as determined on preoperative MRI, plus a 2-cm margin. One year chemotherapy in protocol A consisted of oral trofosfamide (100 mg m^−2^ per day) and oral etoposide (25 mg m^−2^ per day) given once daily for 21 days, repeated after 1 week of rest. In the following protocol (B), patients underwent an intensive synchronous radiochemotherapy regimen soon after surgery, consisting of two courses of induction chemotherapy (cisplatin and etoposide in the first and cisplatin, etoposide and ifosfamide in the second course). This synchronous radiochemotherapy was also used for protocols C and D. In protocol C, vincristine (1.5 mg m^−2^) was added once a week during synchronous radiochemotherapy. In addition, six courses of PEI were given as consolidation chemotherapy every 4 weeks after. In protocol D, patients were either randomised to receive two doses of high-dose methotrexate (5 g m^−2^) before induction radiochemotherapy or to immediately undergo induction radiochemotherapy. Consolidation therapy in this study consisted of lomustine, vincristine and prednisone (every 6 weeks for eight courses).

Response to treatment was defined as previously described (Gnekow 1995; Wolff *et al*, 2000). Follow-up MRI was performed every 3–6 months after radiochemotherapy according to protocol guidelines and when the clinical course suggested disease progression.

### Diagnostic evaluation

The initial diagnostic evaluation for entering the study included a complete physical and detailed neurological examination as well as a brain MRI study with and without gadolinium. Spinal MRI and cytologic examination of CSF were recommended but not mandatory. Histopathologic diagnosis and grading of tumours were performed by local neuropathologists according to the World Health Organization classification of CNS tumours (Kleihues *et al*, 1993). Central neuropathologic review of the local pathological diagnosis was performed in almost all patients at the Neuropathological Reference Centre at the University of Bonn in Germany. The radiological diagnosis of a DIPG was reviewed by the Neuroradiological Reference Centre in Würzburg, Germany. The criteria for DIPG were that the tumour had to be larger than 50% of the pontine diameter. Criteria for HGG were a histopathological diagnosis of a WHO°III or °IV glioma. The extent of surgical resection was documented using the neurosurgeon's report and postoperative contrast-enhanced MRI. Diagnosis of SDD was based on MRI studies and was defined as evidence of linear or nodular contrast-enhancing lesions outside the area of the primary tumour on follow-up MRI studies.

### Statistical analysis

Statistical analyses were performed using the commercially available program SPSS version 12.0; SPSS Inc., Chicago, IL, USA). The *χ*^2^-test was used to compare patient subgroups concerning HGG, DIPG, SDD, age, gender, extent of resection. The Kaplan–Meier method was used to estimate OS rates and median OS time. The 95% confidence interval (CI) was defined by the program as the 95% probability that the calculated OS time can be found within this interval. Differences in OS were analysed with the log-rank test and were estimated as relevant different with a *P*-value of <0.05. Results were updated 30 April 2005.

## RESULTS

### Patient characteristics

During the period studied, 653 patients <21 years old with newly diagnosed HGG or DIPG were registered into the above described database. Of the 653 patients, 368 patients had HGG and 257 had DIPG. We were not able to classify the remaining 28 patients because of the lack of a reference radiological report and not knowing the extent of the tumour. The eligibility criteria for this study were met by 270 patients. Of these, 146 had HGG and 110 had DIPG. Fourteen patients could not be classified into either of these groups because of a lack of clinical data. Forty-six (17%) of the 270 patients developed SDD during the period studied. All patients did not have metastases at primary diagnosis. The clinical features of these patients are summarised in Table 2. The overall incidence of SDD was 12.7% (14/110) for patients with DIPG, and 21.9% (32/146) for patients with HGG.

The median time between diagnosis of the primary tumour and SDD was 8.2 months (range, 2.2 months–6.2 years) for the whole group, 7.2 months (range, 4.6 months–2.2 years) for the DIPG group, and 8.2 months (range, 2.2 months–6.2 years) for the HGG group. In the majority of patients (*n*=33; 71%), metastases occurred soon after initial diagnosis (i.e., within 1 year). In six patients (13%) metastases were observed between 1 and 2 years after initial diagnosis, and in five patients (11%), metastases occurred more than 2 years after diagnosis (no data available for 2 patients, 5%).

CSF examination was initially performed in 11 patients and was positive in two of these patients. Metastases were found at various combinations of anatomic sites in various combinations (Table 3). Extra-CNS metastases were not found.

### Primary treatment and response

The initial treatment that the 46 patients with SDD received is presented in Table 2. The number of patients treated according to protocols A–D was as follows: protocol A, *n*=7 (15%); protocol B, *n*=11 (24%); protocol C, *n*=20 (44%) and protocol D, *n*=8 (17%). Eight weeks after the initiation of therapy, response was evaluated in the 36 patients before the onset of SDD without a complete tumour resection (Table 4), revealing a complete remission in two patients (6%), partial remission in six patients (17%), stable disease in 20 patients (56%) and progressive disease in eight patients (22%). Six of the seven patients who had complete tumour resections were in continuous complete remission at the time of the first response evaluation. Data on remission status were not available for three patients.

Data on the systemic treatment of patients with SDD were available for only 30 patients because this was no further a protocol question in the A–D protocol (Table 3). Unfortunately, data on second surgeries were not uniformly reported.

### OS of patients with and without SDD

Evaluation of all 270 patients for whom SDD data were available did not reveal any difference in OS between patients with and without SDD. The median OS was 1.02±0.14 years (95% CI, 0.75–1.30 years) for patients with SDD and 1.01±0.06 years (95% CI, 0.90–1.12 years) (*P*=0.473) for patients without SDD. For the patients with HGG, we found a survival difference between patients with and without SDD. The median OS of HGG patients with SDD (*n*=32) was 1.02±0.21 years (95% CI, 0.61–1.43 years), and the median OS of HGG patients without SDD (*n*=14) was 1.41±0.28 years (95% CI, 0.86–1.95 years) (*P*=0.0495). In contrast, in the subgroup of patients with DIPG, no differences in median OS were found between patients with and without SDD.

**Figure 1:**
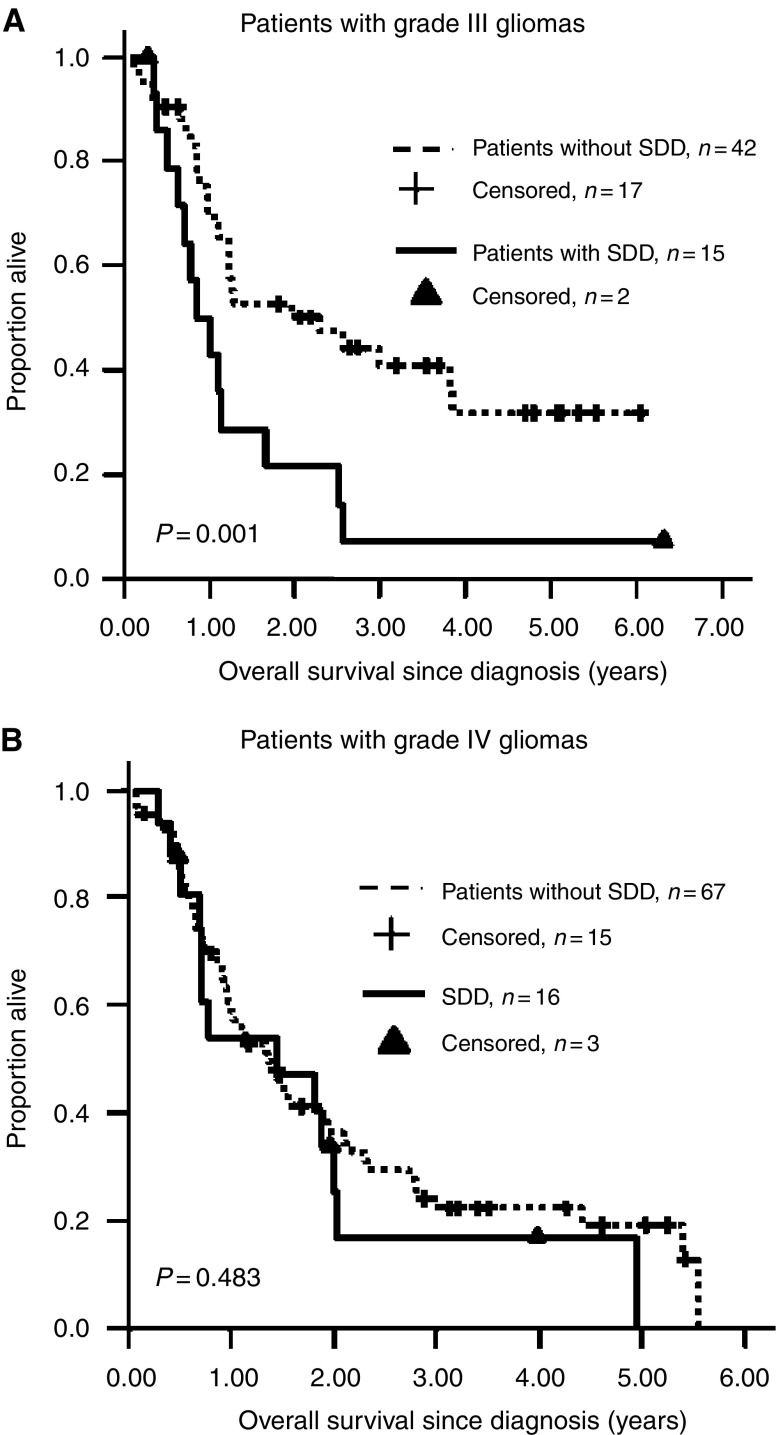
Kaplan–Meier curves for OS of high-grade glioma grade III patients (**A**) and grade IV patients (**B**) with and without SDD.

Further analyses of HGG patients revealed a difference in median OS between patients with (*n*=15; median OS=0.86±0.2 years; 95% CI, 0.46–1.25 years) and without (*n*=42; 2.28±0.95 years; 95% CI, 0.42–4.15 years) SDD among those with grade III tumours (*P*=0.001, Figure 1A) but not among those with grade IV tumours (Figure 1B). Similarly, a difference in OS between HGG patients with (*n*=20; median OS=0.79±0.10 years; 95% CI, 0.59–0.99 years) and without (*n*=73; median OS=1.37±0.15 years; 95 % CI, 0.8–1.66 years; *P*=0.023) SDD was found in younger children (< 13 years) but not in older children (⩾13 years).

**Figure 2:**
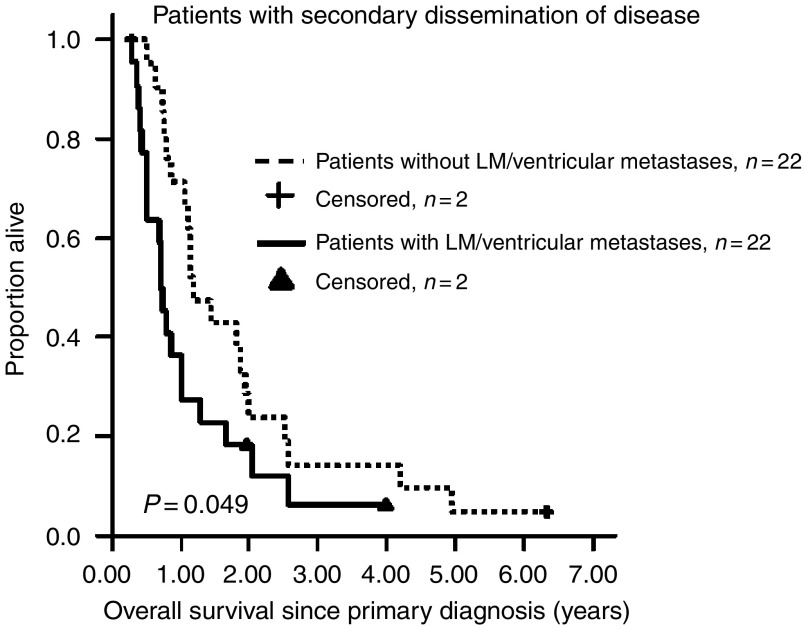
Kaplan–Meier curves for OS of HGG and DIPG patients with secondary disseminated disease with and without leptomenigeal (LM)/ventricular dissemination.

Overall survival was longer for patients with parenchymal metastases (*n*=22; median OS for patients without leptomeningeal/ventricular SDD=1.18±0.19 years; 95% CI, 0.80–1.56 years) compared with patients whose tumours had spread through the CSF (*n*=22; median OS of patients with leptomeningeal/ventricular SDD=0.72±0.05 years; 95% CI, 0.62-0.81 years) (*P*=0.049) (Figure 2). For patients with SDD, the median OS after SDD was 0.27±0.05 years (*n*=44, 95% CI, 0.18–0.37 years) for the HGG and DIPG groups combined. No patient survived longer than 1.8 years after the diagnosis of SDD; the 1-year OS rate after being diagnosed with SDD was 16.7±6.1%. A statistically significant difference in OS was found when the different sites of the intracranial metastases were compared.

## DISCUSSION

This is the largest survey on SDD in paediatric HGG and DIPG patients to date. As expected, the majority (72%) of cases of SDD were detected soon after initial diagnosis (within 1 year). However, there was an interesting small group of five patients who developed SDD more than 2 years after diagnosis, indicating that the biological characteristics of HGG and DIPG tumours are more complex than anticipated.

In our study, SDD did influence OS in patients with HGG, as they had a shorter OS than those without SDD. However, SDD had little impact on the OS of the HGG and DIPG groups combined. Secondary disseminating disease did not reduce OS in the DIPG group, suggesting that rapid local tumour progression was the main cause of the short OS in these patients and that SDD had no additional effect. We conclude, therefore, that in DIPG patients, treatment should primarily focus on controlling local disease.

Among patients with HGG, OS was shorter in patients with SDD in grade III tumours and with age of <13 years. We suggest that HGG patients in these subgroups might benefit from an intensive treatment of metastases, such as spinal or local irradiation or surgery, since tumour progression at the primary site seems to occur less rapidly compared to patients with DIPG.

The overall incidence of SDD in our study was 17% (HGG, 22%; DIPG, 13%), which is in the lower part of the range reported by others (Table 1, Dropcho *et al*, 1987). The highly variable incidence rates of SDD in these other studies might be explained by the fact that they were single-centre-based studies with a limited number of patients, or that they had incomplete CNS staging and different MRI surveillance strategies. In addition, treatment intensity, including high-dose chemotherapy, and post-mortem examinations might have had some impact on the proportion of patients with HGG or DIPG who were diagnosed with SDD in those studies (Table 1). Treatment intensity, in particular, might alter the metastatic potential of HGG and DIPG, thereby affecting the OS in patients with these diseases. Normally data on SDD in HGG or DIPG patients were seldom available because the patients had a short OS, so dissemination did not have time to occur.

Interestingly, in HGG and DIPG patients, dissemination to leptomeningeal and ventricular tumour sites resulted in shorter OS compared with parenchymal metastases; this observation could redirect new treatment approaches. One of these new approaches could be intrathecal administration of chemotherapeutic drugs as it was proposed by Donahue *et al* (1998).

Before initiating new treatment approaches, however, better diagnostic standards, such as craniospinal MRI and mandatory CSF examination, must be implemented in addition to more sensitive methods for screening CSF. Risk factor analysis (e.g., genetic factors to predict SDD in HGG) is another important issue to be addressed in future studies. Even if this retrospective study is the largest survey of patients with SDD, patient numbers are still small. In order to get more experiences with SDD in the group of HGG and DIPG, new protocols should at least plan prospective detailed documentation of SDD. For the first time it was reported that SDD is as a negative prognostic factor in HGG. This finding should still be confirmed by other studies, however, it does suggest a biological relevance of secondary metastases in high-grade glioma, and leads to reconsider intrathecal therapy for these tumours in children.

## Figures and Tables

**Table 1:** Studies reporting on dissemination of high-grade gliomas

**References (no)**	**Histology (n)**	**Total number of patients**	**Site of dissemi-nation**	**% of SDD**	**Interval between primary diagnosis and dissemination median (range)**	**OS after diagnosis of dissemination median (range)**	**Treatment of primary tumour**	**Treatment of dissemination (***n***)**
Arita *et al*, 1994 (5)	GBM (10), AA (12)	157	ST BS, CB	14	20 m (mean) (4–416 weeks)	6 mo (2–41 weeks)	Surgery RT, CT	ith-Ommaya-CT, lumbal irradiation
Dropcho *et al*, 1987 (15)	GBM (13), AA (29), malignant glioma (8)	50	LM	26	7 months		Surgery, CT, RT	ith. interferon focal RT, second surgery
Endo *et al*, 2003 (20)	Cerebellar gliomas GBM (2), AA (*n*=2), anaplastic pilocytic astrocytoma (*n*=1)	5	LM	100	10 months (5–29)	(2.8–4.8 mo)	Surgery, local RT+ACNU	Whole Brain RT+CT (3) VP shunt MTX ith
Grabb *et al*, 1992 (14)	GBM (4), mixed glioma (1), Anaplastic oligodendroglioma (3), AA (2), anaplastic ependymoma (1)	33	LM, V	33	8 mo (1 w–59 months)	4 mo (3 w- 11 mo)	Surgery, CT, irradiation	Conventional CT ,no, High-dose+BM rescue (1) spinal irradiation+CT (2)
Heideman *et al*, 1997 (16)	GBM (*n*=6), AA (*n*=3)	29	LM, SP	31	3 y: 26% risk	ND	CSI	ND
Packer *et al*, 1985 (17)	AA (*n*=34)	23		49		4 months	ND	ND
Saito *et al*, 2003 (22)	AA, GBM	68	All	25	10.3 months (0–31.4)	8.6 months	ND	ND
		17	SP	35	12.7 mo (7–35 mo)	5.6 months		
Vertosick and Selker, 1990 (4)	GBM	11	LM, SP	100	14.1 mo (5–62 mo)	2.8 months (2-7mo)	ND	ND
Donahue *et al*, 1998 (21)	BS gliomas	18	LM	50	9 months (4–17 months)	9 months PFS		
Allen *et al*, 1999 (23)	BS gliomas	29	n.d.	44				
Packer *et al*, 1990, 1993 (18,19)	BS gliomas	35, 48	LM, LM	14, 8	ND	ND	ND	ND
Current study	GBM (20), AA	270	LM +	17	8.2 months	3.2 months	Irradiation	Relapse CT:
	(17), GC-GBM		V				98%	topotecan, temozolomide
	(1), DIPG (8)						SRCT:46%	other CT;
								
	Only pontine gliomas	110		13	7.2 months	3.6 months	CT:91%	RT, surgery: ND
	Only nonpontine gliomas	146		22	8.2 months	3.1 months		

AA, anaplastic astrocytoma; BM, bone marrow; BS, brainstem; CB, cerebellar; CSI, craniospinal irradiation; CT, chemotherapy; DD, disseminated disease; DIPG, diffuse intrinsic pontine glioma; GBM, glioblastoma multiforme; GC-GBM, giant cell GBM; ith, intrathecal; LM, leptomeningeal; mo, months; nd, no data available; PFS, progression-free survival; RT, radiotherapy; SRCT, simultaneous radiochemotherapy (cisplatin; etoposide; ifosfamide; vincristine; methotrexate); SP, spinal; ST, supratentorial; V, ventricular; VP, ventriculoperitoneal; w, weeks; y, year.

**Table 2:** Clinical characteristics of patients with secondary metastases (*n*=46) and without SDD (*n*=224)

	**Patients with SDD**		**Patients without SDD**	
**Characteristics**	***n***	**%**	***n***	**%**
*Gender*				
Male	27	58.7	115	51.3
Female	19	41.3	109	48.7
Median age at primary diagnosis	10.2 years (0.1–19.1 years)		9.2 years (0.1–21 years)	
				
*Primary tumour site*				
Cerebral hemispheres	14	30.4	53	23.7
Pons/brainstem	14	30.4	106	47.3
Thalamus/basal ganglia	6	13.0	24	10.7
Cerebellum	5	10.9	5	2.2
Ventricles	2	4.3	6	2.7
Medulla	1	2.2	1	0.4
Pineal region	1	2.2	1	0.4
Hypothalamus	0	0	2	0.9
Spinal cord	1	2.2	4	1.8
Gliomatosis cerebri	2	4.3	11	4.9
Brain NOS	0	0	11	4.9
				
*Diagnosis*				
Glioblastoma multiforme grade IV	20	43.5	76	33.9
Giant cell glioblastoma grade IV	1	2.2	2	0.9
Anaplastic astrocytoma grade III	17	37.0	63	28.1
Oligoastrocytoma/-dendroglioma grade III	0	0	5	22.3
Gliosarcoma grade IV	0	0	2	0.9
Astroblastoma/xantoastrocytoma grade III	0	0	3	1.3
Astrocytoma WHO grade I/II (DIPG)	2	4.3	16	7.1
DIPG, only radiologic diagnosis	6	13.0	57	25.4
				
*Spinal MRI* at primary diagnosis	10/39*	25.6	nd	
**CSF** positive at primary diagnosis	2/11*	18.2	3/33	9.1
				
*Resection of the primary tumour* (*n*=222)				
Complete resection	7	15.2	33	14.9
Subtotal resection (⩾95% of the tumor removed)	9	19.6	24	10.8
Partial resection (< 95% of the tumor removed)	9	19.6	39	17.7
				
*Biopsy*	15	32.6	75	33.8
*No surgery*	6	13.0	51	22.9
				
*Irradiation* of the primary tumour (*n*=221)				
Yes	45	97.8	201	90.9
No	1	2.2	20	9.1
				
*Simultaneous radiochemotherapy* of the primary tumor (*n*=220)				
Yes	21	45.7	95	43.2
No	25	54.3	125	56.8
				
*Chemotherapy* of the primary tumour (*n*=221)				
Yes	42	91.3	204	92.3
No	4	8.7	17	7.7

MRI=magnet resonance imaging; CSF=cerebrospinal fluid; DIPG=diffuse intrinsic pontine glioma; NOS=no other specification.

* Data were available for only 39 or 11 patients.

**Table 3:** Characteristics of secondary dissemination

	**Number of patients (%)**		
**Characteristics**	**Whole group**	**HGG**	**DIPG**
Median time from primary diagnosis to secondary dissemination	8.2 months	8.2 months	7.2 months
**Parenchymal metastasis**	(*n*=46)	(*n*=32)	(*n*=14)
Supratentorial	16 (34.8%)	11 (34.4%)	5 (35.7%)
Supra-+infratentorial	9 (19.6%)	6 (18.8%)	3 (21.4%)
Infratentorial	3 (6.5%)	2 (6.3%)	1 (7.1%)
Spinal	5 (10.9%)	4 (12.5%)	1 (7.1%)
Cerebral+spinal	3 (6.5%)	0	3 (21.4%)
Leptomeningeal dissemination	10 (21.7%)	9 (28.1%)	1 (7.1%)
**Chemotherapy of disseminated disease**	(*n*=30)	(*n*=22)	(*n*=8)
Topotecan	7 (23.3%)	4 (18.2%)	3 (38%)
Temozolomide	11 (36.7%)	9 (40.9%)	2 (25%)
Valproic acid	1 (3.3%)	1 (4.5%)	0 (0%)
Other chemotherapy	9 (30.0%)	6 (27.3%	3 (38%)
No chemotherapy	2 (6.7%)	2 (9.1%	0 ((0%)
**Irradiation of disseminated disease**	2	2	0
**Second surgery of disseminated disease**	ND	ND	ND
**No treatment of disseminated disease**	2	2	0
Median OS after diagnosis of secondary dissemination (months)	3.2	3.1	3.6

ND=no data available; DIPG=diffuse intrinsic pontine gliomas; HGG=high-grade gliomas; OS=overall survival.

**Table 4:** Comparison of response in HGG and DIPG patients with and without secondary dissemination 8 weeks after primary treatment

**Response to primary treatment**	**Before SDD**		**Without SDD**	
CCR	6/7*	14.3%	22/33	11%
Response (complete and partial)^a^	8/36	22.3%	37/170	21.7%
Stable disease^a^	20/36	55.6%	84/170	49.4%
Progressive disease^a^	8/36	22.3%	49/170	28.8%

CCR=continuous complete remission after complete resection; DIPG=diffuse intrinsic pontine gliomas; HGG=high-grade gliomas; SDD=secondary disseminating disease.

* Six of seven patients available for this analysis.

a In patients without complete tumour resection.
